# Deciphering host–virus interaction networks in ALV infection: an integrative multi-omics perspective

**DOI:** 10.3389/fimmu.2026.1855941

**Published:** 2026-06-30

**Authors:** Junliang Xia, Weiding Chen, Xiaoli Zhou, Tao Xu, Guodong Mo, Xiquan Zhang

**Affiliations:** 1 College of Animal Science, Department of Animal Genetics, Breeding and Reproduction, South China Agricultural University, Guangzhou, China; 2 Key Laboratory of Agricultural Animal Genomics and Molecular Breeding of Guangdong Province, Key Laboratory of Chicken Genetics, Breeding and Reproduction of Ministry of Agriculture and Rural Affairs, Guangzhou, China; 3 National Key Laboratory of Pig and Poultry Breeding, Guangzhou, China; 4 Guangxi Vocational University of Agriculture, Nanning, Guangxi, China

**Keywords:** avian leukosis virus, CRISPR screening, host-virus interactions, innate immunity, molecular phenotypes, multi-omics integration

## Abstract

Avian leukosis virus (ALV) remains a major threat to the poultry industry due to its ability to establish persistent infection, induce immunosuppression, and promote tumorigenesis. Despite progress in eradication programs, effective control is hindered by subclinical infection, vertical transmission, and rapid viral evolution. A key limitation lies in the complexity of host–virus interactions, which are governed by multi-layered regulatory processes that cannot be fully resolved by studies focusing on single genes, pathways, or omics layers. In addition, the lack of robust and dynamic phenotypic indicators further constrains the dissection of resistance mechanisms. Recent advances in multi-omics technologies provide an opportunity to overcome these challenges by capturing coordinated changes across genomic, transcriptional, proteomic, and epigenetic levels. Integrating these data with molecular phenotypes, such as expression quantitative trait loci (eQTLs) and protein quantitative trait loci (pQTLs), enables the linking of genetic variation to functional immune responses. Furthermore, CRISPR-based functional screening offers a systematic approach for validating candidate host factors and identifying key regulators of viral replication and immune modulation. In this review, we summarize the epidemiology and molecular biology of ALV, outline current understanding of host–virus interaction networks with an emphasis on innate immune responses, and highlight how integrative multi-omics combined with functional genomics can advance the identification of critical host determinants. This framework provides a systems-level perspective for deciphering ALV–host interactions and supports the development of more effective antiviral strategies.

## 1 Introduction

Avian leukosis virus (ALV), a member of the genus alpharetrovirus, remains a persistent threat to the global poultry industry due to its ability to establish lifelong infection, induce immunosuppression, and promote tumorigenesis (1). Despite partial success of eradication programs in selected regions, ALV continues to circulate widely, particularly in environments with limited biosecurity, driven by vertical transmission, subclinical infection, and rapid viral evolution (2). Over the past decades, substantial progress has been made in defining the molecular biology of ALV, including its genomic organization, replication cycle, and oncogenic mechanisms (3–5). However, a comprehensive understanding of host–virus interaction networks is still lacking, which has constrained the identification of resistance-associated factors and limited progress in antiviral strategies (6).

Existing studies have primarily focused on individual host genes or isolated signaling pathways involved in ALV replication and immune responses. While these findings have provided important mechanistic clues, they remain fragmented and fail to capture the coordinated, multi-layered regulatory architecture of host immunity (6). Consequently, the network-level logic governing antiviral responses, immune dysregulation, and viral adaptation remains poorly defined. In addition, host–ALV interactions are shaped by complex and dynamic regulation across multiple biological layers, including genomic variation, transcriptional dynamics, protein activity, and epigenetic modifications. The predominantly subclinical nature of ALV infection further complicates phenotypic evaluation, as commonly used indicators such as viremia are often temporally variable, noisy, and tissue-specific (7), thereby limiting the accurate dissection of host immune responses during infection.

Recent advances in high-throughput technologies—including genomics, transcriptomics, proteomics, and epigenomics—have provided powerful tools to systematically investigate host–virus interactions (8, 9). However, most studies remain confined to single-omics layers, restricting the ability to resolve cross-level regulatory mechanisms (10). The integration of molecular phenotypes, such as expression quantitative trait loci (eQTLs) and protein quantitative trait loci (pQTLs), offers an effective framework to bridge genetic variation with functional immune outcomes, thereby improving the resolution of host factor identification (11, 12). In parallel, CRISPR-based functional screening technologies have emerged as scalable platforms for high-throughput perturbation and validation of host dependency factors and immune regulatory genes (13–15). The integration of multi-omics analyses with CRISPR-based perturbation thus provides a robust strategy to identify key regulatory nodes governing ALV–host interactions.

In this review, we summarize the epidemiological characteristics and molecular biology of ALV, and provide a systematic overview of innate immune recognition mechanisms and host factors involved in viral replication control. We further highlight recent advances in multi-omics approaches for dissecting host–virus interaction networks. Finally, we propose an integrative systems framework that combines multi-omics profiling with CRISPR-based functional validation to identify critical regulators of ALV infection, aiming to advance mechanistic understanding of host–virus interactions and inform future antiviral strategies in poultry.

## 2 Overview of ALV

### 2.1 Classification and epidemiology of ALV

Based on antigenic differences in the viral envelope glycoprotein (gp85) and receptor usage, avian leukosis viruses (ALVs) infecting chickens are classified into several subgroups, including A, B, C, D, E, J, and K (16). Among these, subgroups A, B, C, D, J, and K are exogenous viruses with varying degrees of pathogenicity, whereas subgroup E represents endogenous ALVs that are integrated into the host genome and are generally non-pathogenic, although they may contribute to viral evolution through recombination events (17). Among the exogenous subgroups, ALV-J is considered the most pathogenic and economically important variant, characterized by strong tumorigenic potential and efficient transmission (18, 19). In contrast, ALV-K is a relatively recently identified subgroup, and its pathogenicity and epidemiological characteristics remain to be fully elucidated (20). Currently, ALV-J is regarded as one of the predominant prevalent subgroups worldwide, although subgroups A and B continue to persist or re-emerge in certain regions (16).

With the implementation of rigorous eradication programs, including routine testing and culling of infected breeding flocks, many developed countries have largely controlled the spread of exogenous ALVs. However, in developing regions such as Egypt, Pakistan, and China, ALV remains endemic due to limitations in biosecurity measures and breeding management systems (21–23). In addition to vertical transmission, alternative sources of infection have also been reported, including contamination of commercial vaccines with replication-competent ALV (21, 24)and the potential role of backyard or pet birds as viral reservoirs (25). Epidemiological studies in China since 2010 indicate that subgroups A, B, K, and J are currently circulating in local chicken populations, with ALV-J being the dominant subtype (26). Although the prevalence of ALV has declined due to improved eradication programs, the virus persists in indigenous breeds and small-scale production systems (27). Moreover, ALV continues to evolve through host–virus interactions, with genetic variation in ALV-J potentially altering its replication efficiency and pathogenicity (2). Collectively, these findings highlight that ALV remains a persistent and evolving threat, underscoring the importance of long-term surveillance and the identification of host factors that may enable the resistance in chicken.

### 2.2 Molecular structure and life cycle of ALV

**Figure 1:**
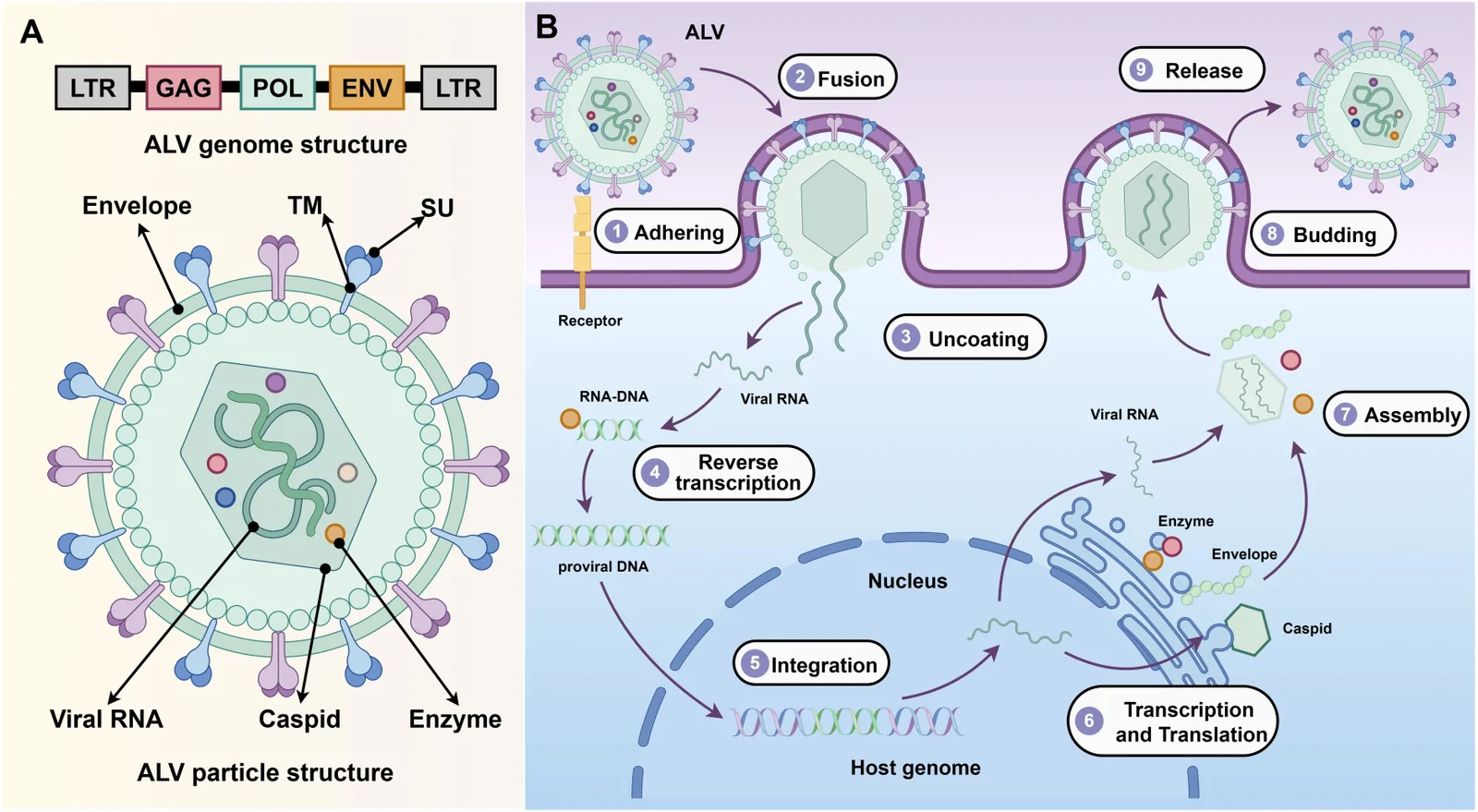
Molecular structure and life cycle of ALV. Schematic illustration of the genomic organization, virion structure, and replication cycle of ALV. **(A)** The ALV genome consists of long terminal repeats (LTRs) flanking the gag, pol, and env genes. The virion is an enveloped particle containing viral RNA, nucleocapsid, and associated enzymes, with SU and TM glycoproteins embedded in the lipid envelope. **(B)** The ALV life cycle includes attachment (1), fusion (2), uncoating (3), reverse transcription (4), integration (5), transcription and translation (6), assembly (7), budding (8), and release (9).

ALV is an enveloped, single-stranded positive-sense RNA virus with a genome of approximately 7.2–7.8 kb, organized as 5′-LTR-gag-pol-env-3′-LTR (28). The virion exhibits a typical retroviral architecture, consisting of an inner genomic RNA-protein complex, a surrounding capsid layer, and a lipid envelope embedded with surface (SU) and transmembrane (TM) glycoproteins (**Figure 1A**). The gag, pol, and env genes encode structural proteins, replication enzymes, and envelope glycoproteins, respectively.

The viral life cycle follows a typical retroviral replication strategy, including receptor-mediated entry, reverse transcription, integration into the host genome, and subsequent transcription, translation, assembly, and release (29) (**Figure 1B**). A defining feature of ALV infection is the stable integration of proviral DNA, enabling persistent infection and long-term interaction with host cellular systems (30). Importantly, each stage of the viral life cycle provides potential regulatory nodes that can be modulated by host factors, thereby influencing viral replication efficiency and pathogenic outcomes (4). These characteristics highlight that ALV infection is governed not only by viral determinants but also by complex host-virus interactions.

## 3 Host–virus interaction networks in ALV infection

### 3.1 Receptor-mediated viral entry

Infection by ALV is initiated through the interaction between viral envelope proteins and specific host cell receptors, representing the first step of host-virus interaction. To date, receptors corresponding to all major ALV subgroups have been identified. Subgroups A and K utilize the receptor Tva (31, 32), subgroup C uses Tvc (33), subgroups B, D, and E share the receptor Tvb (34, 35), and subgroup J specifically recognizes the sodium/hydrogen exchanger 1 (NHE1) (36). Importantly, variants of these receptor genes can determine host susceptibility or resistance to ALV infection (37–39).

Recently, CRISPR/Cas9-mediated modification of the Tva receptor has been shown to confer resistance to ALV-A/K infection. However, disruption of this receptor also affects vitamin B12 transport, which compromises the long-term viability of the edited chickens (40). In contrast, targeted mutations in the NHE1 receptor can effectively block ALV-J entry without significantly affecting host survival (41). Beyond genome editing, resistance can also be achieved by exploiting naturally occurring resistant alleles. For example, by genotyping chickens carrying resistance-associated loci such as Tva^r2^ and Tvb^r3^, and subsequently performing selective breeding combined with primordial germ cell (PGC) transplantation, researchers have successfully generated chimeric chickens exhibiting resistance to both ALV-A/K and ALV-B/D/E while maintaining normal development (37). Nevertheless, the rapid evolution and adaptability of ALV allow the virus to overcome single-site genetic barriers over time (42). These observations highlight that the evolutionary arms race between viruses and hosts is unlikely to be resolved by single nucleotide or amino acid changes alone. Moreover, these findings emphasize the importance of further elucidating the immune regulatory mechanisms underlying host-virus interactions.

### 3.2 Innate immune responses to ALV infection

**Figure 2:**
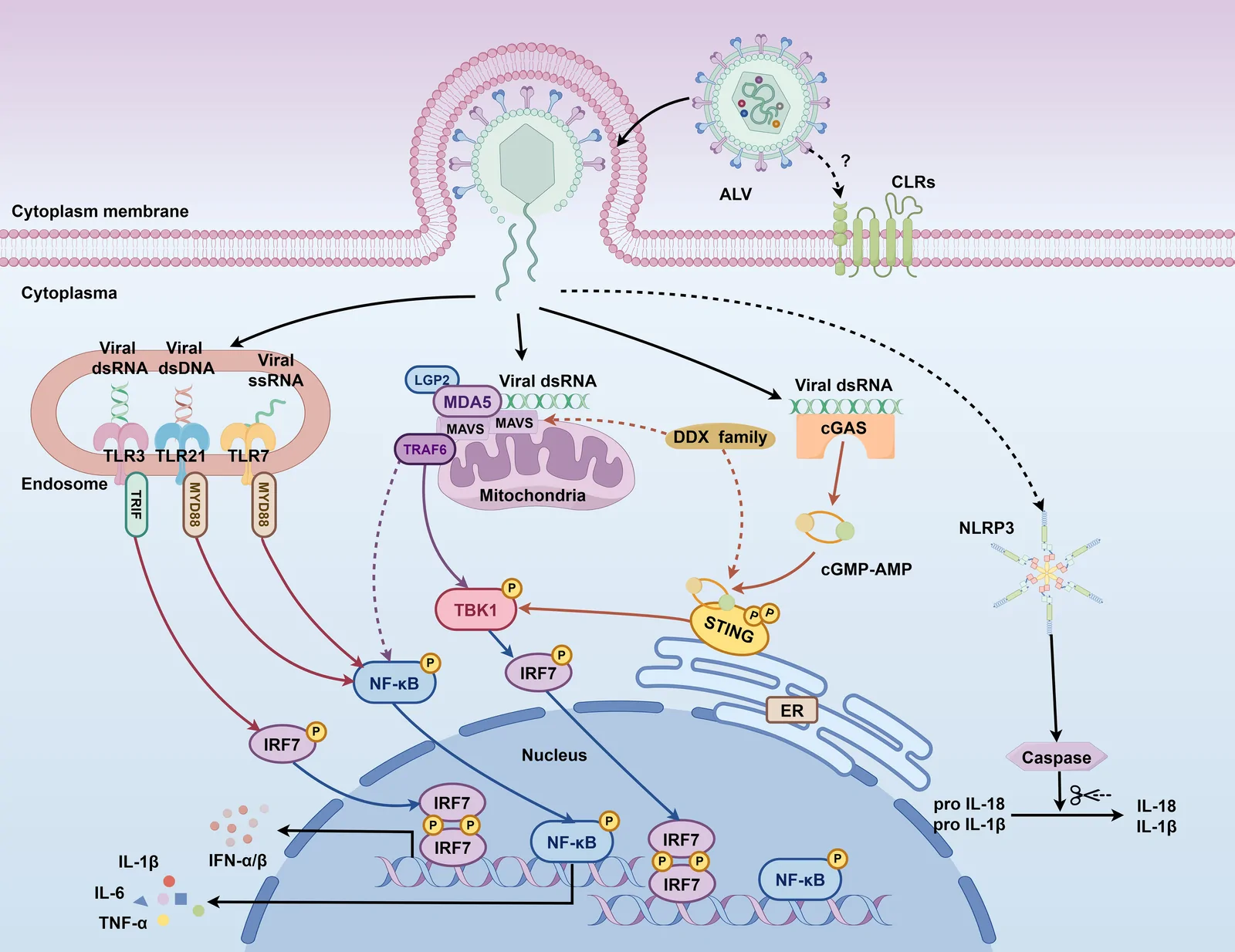
Innate immune recognition pathways and signaling cascades during ALV infection. Upon entry, ALV-derived nucleic acids are sensed by diverse PRRs. Endosomal TLR3, TLR7, and TLR21 signal via TRIF or MyD88 to activate NF-κB and interferon regulatory factor 7 (IRF7). In the cytoplasm, the RLR pathway (MDA5 and LGP2) signals through MAVS, while cGAS senses viral dsDNA. These pathways converge on TBK1, leading to phosphorylation and nuclear translocation of IRF7. Activated IRF7 and NF-κB induce the expression of type I interferons and pro-inflammatory cytokines. In parallel, ALV infection activates the NLRP3 inflammasome–caspase cascade, resulting in maturation and secretion of IL-1β and IL-18. Solid arrows indicate experimentally supported interactions, whereas dashed arrows represent putative or less well-defined mechanisms.

Avian leukosis virus (ALV), an alpharetrovirus, undergoes a replication process involving RNA intermediates and integrated DNA forms, both of which can be detected by the host innate immune system. Pattern recognition receptors (PRRs) act as key sensors of viral nucleic acids and play essential roles in initiating antiviral immune responses. In chickens, several major PRR families have been identified, including Toll-like receptors (TLRs), RIG-I-like receptors (RLRs), NOD-like receptors (NLRs), and C-type lectin receptors (CLRs) (43). These receptor families differ in their cellular localization, ligand recognition patterns, and downstream signaling pathways, thereby contributing to a multilayered innate immune defense against ALV infection (**Figure 2**).

#### 3.2.1 PRR-mediated recognition of ALV and activation of innate immunity

TLRs are membrane-bound or endosomal receptors involved in early viral sensing. Among chicken TLRs, TLR7 appears to be particularly relevant to ALV infection because it recognizes single-stranded RNA (ssRNA), which corresponds to RNA intermediates generated during viral replication (43, 44). ALV-J infection has been shown to significantly upregulate TLR7 expression and activate the MyD88-dependent signaling pathway, leading to the activation of NF-κB and IRF7 and the subsequent induction of type I interferons (IFN-I) and pro-inflammatory cytokines (44, 45). TLR3 may also participate in ALV sensing by recognizing double-stranded RNA (dsRNA) intermediates generated during replication, although its role appears more limited (46, 47). Notably, ALV-J infection has been reported to suppress the expression of TLR3 and TLR4 in dendritic cells, accompanied by reduced production of cytokines such as IL-4, IL-6, and IL-8 (48). This contrasts with other RNA viruses, such as avian influenza virus (AIV), which typically induce TLR3 activation, suggesting that ALV may evade host immunity by actively modulating TLR signaling pathways. TLR21, the functional homolog of mammalian TLR9, recognizes CpG DNA motifs. However, given that ALV primarily exists as an RNA virus, direct evidence supporting a major role of TLR21 in ALV infection is limited, and its function may be restricted to secondary immune regulation (49, 50). Overall, TLRs primarily contribute to the early detection of viral RNA and the initiation of MyD88 or TRIF dependent antiviral signaling cascades.

RLRs are cytoplasmic RNA sensors that play a central role in antiviral immunity against RNA viruses (51). Unlike mammals and waterfowl, chickens lack RIG-I and instead rely on MDA5 and LGP2 (52). MDA5 serves as the primary sensor for viral RNA, recognizing long dsRNA structures and signaling through mitochondrial antiviral signaling protein (MAVS), leading to the activation of TBK1 and IKKϵ and subsequent induction of IRF7 and NF-κB. This results in robust production of IFN-I and downstream interferon-stimulated genes (ISGs) (53). During ALV infection, RNA intermediates can be detected by MDA5, thereby activating antiviral responses (54, 55). Knockdown of chMDA5 significantly reduces IFN-β expression and the transcription of antiviral genes such as interferon β promoter stimulator 1 (IPS-1), 2′-5′-oligoadenylate synthetase (OAS), and Myxovirus resistance (Mx1), highlighting its essential role in host defense (55). Laboratory of genetics and physiology 2 (LGP2), although lacking caspase activation and recruitment domains (CARD) required for independent signaling, functions as a regulator of MDA5 and enhances its RNA recognition capacity (56, 57). Waterfowl, particularly ducks, are easily infected with a wide range of avian influenza virus (AIV) subtypes and typically remain asymptomatic upon infection, whereas chickens are highly susceptible and often develop severe or lethal disease (43). Interestingly, DF-1 cells has been shown to enhance resistance to AIV after RIG-I knock-in, highlighting its importance in antiviral innate immunity (58). Thus, the absence of RIG-I in chickens may reduce the efficiency of viral RNA recognition, thereby weakening early immune responses and potentially contributing to the persistence of infections such as ALV.

NLRs are cytoplasmic sensors primarily involved in inflammation and inflammasome formation. Viral infections can activate the NLRP3 inflammasome, leading to caspase-1 activation and the maturation of IL-1β and IL-18 (59). However, studies on NLRs in chickens remain limited, and their roles in ALV infection are not well defined (43). Given that ALV induces chronic infection and immunosuppression, it is plausible that the virus modulates NLR-associated pathways to alter the inflammatory microenvironment, thereby indirectly promoting viral persistence.

CLRs recognize carbohydrate structures and participate in pathogen binding, internalization, antigen presentation, and immune regulation (60–62). As an enveloped virus displaying glycoproteins such as gp85, ALV may interact with CLR-related recognition systems during attachment or immune modulation. Nevertheless, current evidence suggests that CLRs play an auxiliary rather than central role in ALV infection, and their precise functions require further investigation.

Beyond these classical PRR families, several non-canonical nucleic acid sensors also contribute to antiviral immunity. Members of the DEAD-box RNA (DDX) helicase family, including DDX1, DDX21, and DHX36, have been shown in mammals to recognize viral RNA analogs and interact with MAVS, LGP2, and RIG-I to regulate antiviral signaling (63). In addition, the cGAS–STING pathway represents a key mechanism for cytosolic DNA sensing. cGAS detects double-stranded DNA and produces cyclic GMP-AMP (cGAMP), which activates STING and subsequently triggers TBK1-dependent activation of IRF7 and NF-κB, leading to IFN-I production (64–66). Although ALV is an RNA virus, its reverse transcription process generates DNA intermediates that may be recognized by this pathway. Notably, ALV p15 can interact with IRF7 and antagonize cGAS–STING signaling, thereby promoting viral replication (67). Collectively, these PRR-mediated pathways constitute a multilayered sensing system that detects ALV infection and initiates antiviral responses. However, ALV has evolved multiple strategies to modulate or evade these pathways, contributing to its ability to establish persistent infection (68).

#### 3.2.2 Host factor-mediated interaction networks

Downstream of PRR activation, a diverse array of host factors regulates ALV replication and host responses. Rather than acting as isolated effectors, these factors form a multi-layered regulatory network, with representative host factors summarized in **Table 1**. Importantly, these host factors function in a bidirectional manner, either restricting viral replication or being exploited by the virus to facilitate infection, thereby constituting the core of host–virus interaction networks.

**Table 1:** Representative host factors involved in ALV infection and their functional classification.

Gene	Functional category	Mechanism	Subgroup	Cell type	Target/Progress	Role in viral life cycle	Reference
ZAP	Direct antiviral	Binds viral envelope protein to inhibit replication	ALV-J	DF-1	SU protein	Inhibition	(69)
MARCH2	Direct antiviral	Promotes ubiquitination and proteasomal degradation	ALV-A	DF-1	gp85 (K282)	Inhibition	(70)
NCL	Direct antiviral	Suppresses transcription by binding viral regulatory regions	ALV-K	DF-1/HEK293T	LTR/U3	Inhibition	(71)
THY1	Direct antiviral	Induces proteasomal degradation of receptor	ALV-J	DF-1	NHE1	Inhibition	(72)
gga-miR-1650	Direct antiviral	Binds viral RNA to suppress protein expression	ALV-J	DF-1/HEK293T	Gag (5′UTR, p27)	Inhibition	(73)
lnc-LTR5B	Direct antiviral	Disrupts host–virus RNA interaction	ALV-J	CEF/DF-1	Bip-viral RNA	Inhibition	(74)
Cables1	Direct proviral	Promotes polyubiquitination of p15	ALV-J	DF-1	p15	Promotion	(75)
SERBP1	Direct proviral	Enhances LTR activity via U3 binding	ALV-K	DF-1/HEK293T	LTR/U3	Promotion	(76)
MIF	Direct proviral	Recruited by Gag proteins	ALV-J	DF-1	Gag protein	Promotion	(77)
ACSL1	Immune regulation	Enhances IFN response and induces apoptosis	ALV-J	DF-1/MDM	IFN signaling/cell apoptosis	Inhibition	(78)
CSF3	Immune regulation	Activates IFN and NF-κB pathways	ALV-J	DF-1	IFN signaling/Inflammation	Inhibition	(79)
TRIM25	Immune regulation	Enhances MDA5-mediated IFN response	ALV-A	DF-1	RLR pathway	Inhibition	(80)
PRL	Immune regulation	Promotes inflammatory responses	ALV-J	DF-1	Inflammation	Inhibition	(81)
TLR3	Immune regulation	Activates TLR3 signaling and IFN production	ALV-J	DF-1/HD11/ Primary spleen cell	TLR pathway	Inhibition	(82)
SOCS3	Immune regulation	Suppresses JAK/STAT signaling	ALV-J	DF-1	IFN pathway	Promotion	(83)
GSK2β	Immune regulation	Regulates innate immune responses	ALV-J	DF-1	Immune signaling	Promotion	(84)
UBE2J1	Immune regulation	Inhibits STAT3/IRF1 signaling	ALV-A	DF-1	IFN pathway	Promotion	(85)
CH25H	Cellular process regulation	Induces autophagy via 25HC production	ALV-J	DF-1/MDM	Lipid metabolism/Autophagy	Inhibition	(86)
GH	Cellular process regulation	Regulates proliferation and apoptosis via PI3K/Akt	ALV-J	DF-1	Cell cycle/survival	Inhibition	(87)
GREG1	Cellular process regulation	Induces mitophagy and apoptosis	ALV-J	DF-1	Mitochondrial function	Inhibition	(88)
BCL11B	Cellular process regulation	Promotes apoptosis of infected cells	ALV-J	DF-1	Cell apoptosis	Inhibition	(89)
TRIM45	Cellular process regulation	Facilitates apoptosis	ALV-J	DF-1	Cell apoptosis	Inhibition	(90)
gga-miR-148a-5p	Cellular process regulation	Inhibits cell cycle progression	ALV-J	CEF/DF-1	PDPK1	Inhibition	(91)
gga-miR-375	Cellular process regulation	Inhibits proliferation and promotes apoptosis	ALV-J	DF-1/CHO	YAP1	Inhibition	(92)
CTHRC1	Cellular process regulation	Alters protein localization	ALV-J	DF-1	Host protein localization	Promotion	(93)
RRM2	Cellular process regulation	Interacts with p27 and activates Wnt/β-catenin pathway	ALV-J	DF-1/CEF/ HEK293T	p27	Promotion	(94)
TCP1	Cellular process regulation	Inhibits autophagy via AKT activation	ALV-J	DF-1	Autophagy	Promotion	(95)

DF-1, chicken embryo fibroblast cell line; CHO, Chinese hamster ovary cell line; CEF, Primary chicken embryo fibroblast; HEK293T, human embryonic kidney 293T cell; HD11, hen dmacrophage 11.

**Figure 3:**
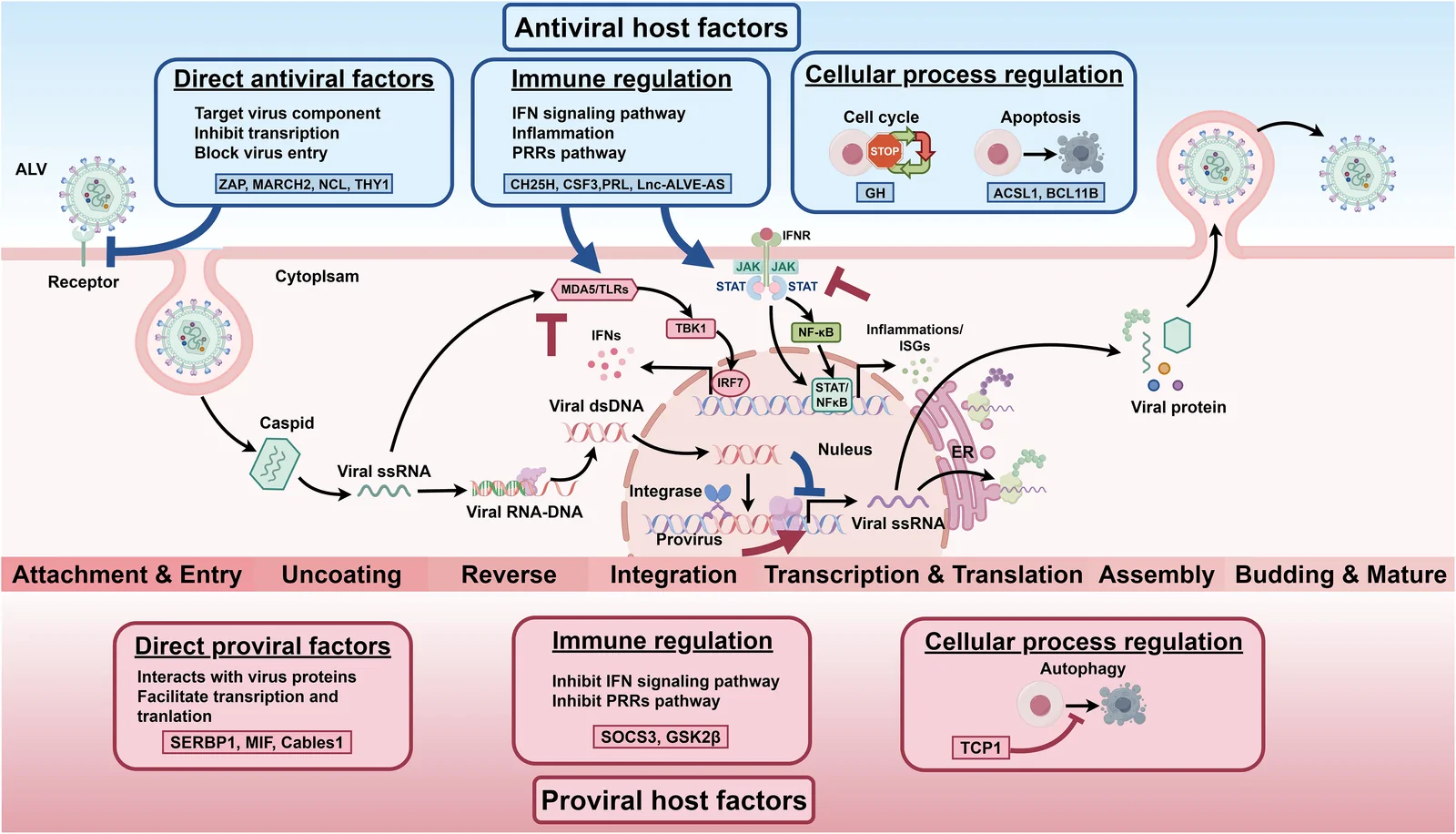
Schematic representation of the ALV replication cycle and its regulation by host factors. This schematic depicts the ALV life cycle and the multilayered host regulatory network engaged during infection. PRR-mediated innate immune sensing is shown as the primary antiviral defense, alongside host factors identified to date that participate in host-virus interactions. These factors function across three major regulatory layers: direct targeting of viral replication processes, immune regulation, and cellular process regulation. Arrows indicate activation or promotion, whereas T-bars indicate inhibition or blockade.

Functionally, host factors can be broadly categorized into three interconnected layers (**Figure 3**). First, direct regulators act on specific stages of the viral life cycle. Antiviral factors inhibit viral entry, reverse transcription, or transcription by targeting viral components or regulatory elements, whereas proviral factors enhance viral replication by stabilizing viral proteins or promoting viral gene expression, for example through interactions with viral structural proteins or long terminal repeats (LTRs) (69, 73). Second, immune modulators regulate signaling pathways such as IFN and NF-κB. In this layer, antiviral factors amplify innate immune responses, leading to increased production of IFN-I and interferon stimulating genes (ISGs), whereas proviral factors attenuate these responses by suppressing key signaling pathways, such as JAK/STAT or IRF-mediated transcription (80, 83). Notably, viral proteins actively participate in this process by interfering with host signaling components, thereby weakening antiviral immunity. Third, cellular regulators influence fundamental processes such as apoptosis, autophagy, metabolism, and cell cycle progression (86). These processes can exert antiviral effects by limiting viral replication or promoting infected cell clearance, but can also be reprogrammed by ALV to create a cellular environment favorable for viral propagation (95).

These functional layers are highly interconnected, and individual host factors often participate in multiple regulatory processes across different layers (96). Consequently, host-virus interactions are not linear but involve complex feedback and crosstalk between antiviral restriction and proviral exploitation. The outcome of infection is therefore determined by the dynamic balance between these opposing forces within the regulatory network. Given the complexity and interconnectivity of these regulatory layers, systematic approaches such as multi-omics analyses are required to identify key host factors and uncover the molecular basis of resistance.

## 4 From single-omics to integrative analysis: dissecting host immune responses to ALV infection

With the rapid development of high-throughput technologies, research on avian leukosis virus (ALV) has shifted from identifying individual host factors to characterizing coordinated host immune responses across multiple regulatory layers. Most existing studies rely on single-omics approaches, which, although informative, provide fragmented insights into host–virus interactions. In reality, ALV infection involves dynamic and interconnected regulation at the genetic, transcriptional, protein, and epigenetic levels. Therefore, a comprehensive understanding of host antiviral immunity requires integrative strategies that link these layers and reconstruct the regulatory networks governing immune activation, modulation, and viral evasion.

### 4.1 Genomics: linking genetic variation to host antiviral immune responses

**Figure 4:**
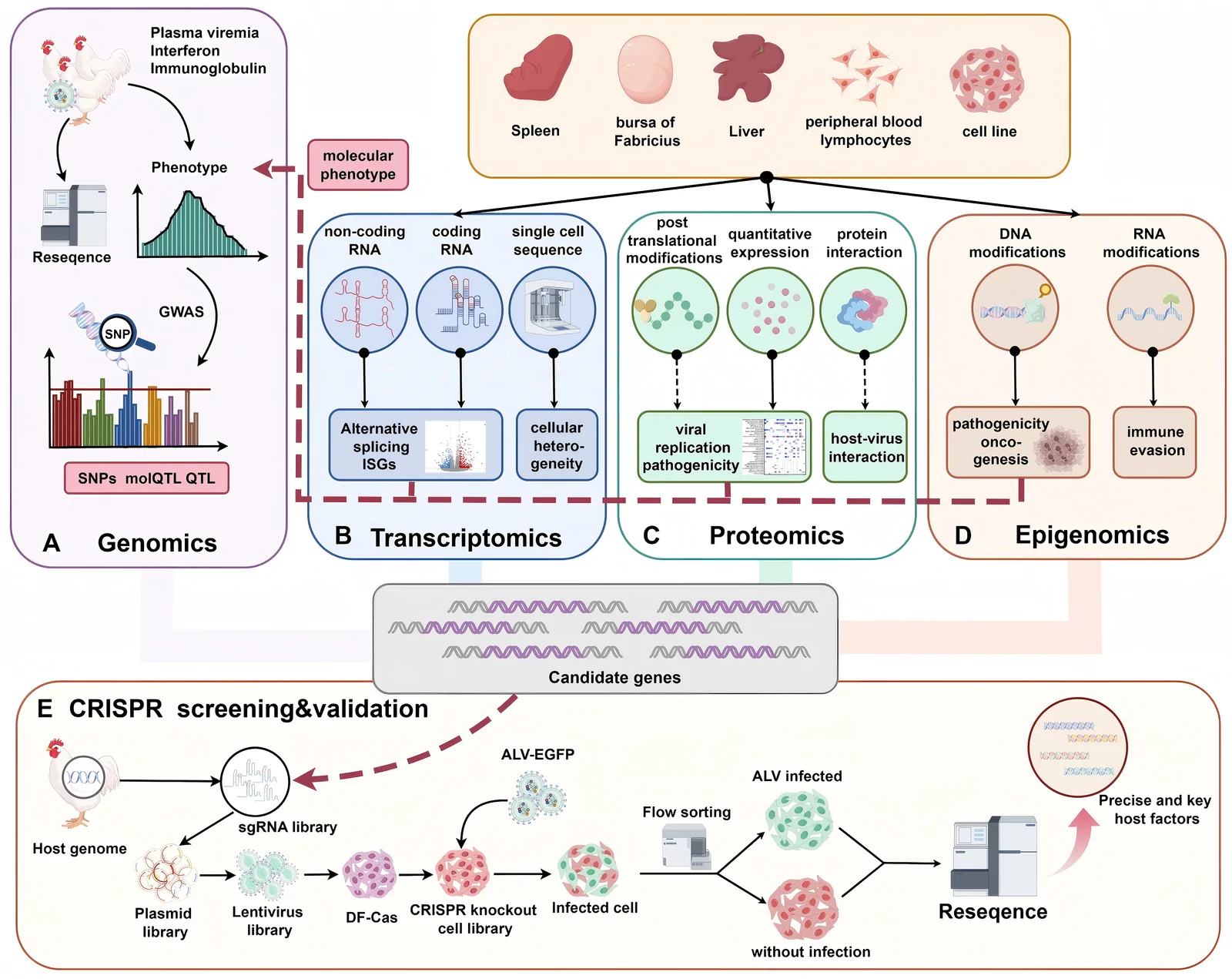
Integrated multi-omics framework for dissecting host–ALV interactions. This schematic summarizes current research strategies for dissecting host-ALV interactions using genomics **(A)**, transcriptomics **(B)**, proteomics **(C)**, epigenomics **(D)**, and CRISPR-based screening **(E)**. Solid arrows indicate established findings and experimentally validated relationships, whereas dashed arrows represent proposed or yet-to-be-explored directions. Together, this framework highlights the progression from multi-omics discovery to functional validation in identifying key host factors regulating ALV infection.

Genomic variation shapes host susceptibility to ALV infection by influencing the strength, timing, and coordination of antiviral immune responses. Genome-wide association studies (GWAS) have identified multiple loci associated with viral load and disease outcomes, many of which are enriched in immune-related pathways, suggesting that ALV resistance is a polygenic trait involving diverse biological processes (97, 98). However, the subclinical and dynamic nature of ALV infection complicates phenotype definition, as commonly used indicators such as viremia exhibit strong temporal variability (7) (**Figure 4A**). To improve the resolution of genetic analyses, molecular phenotypes—including gene expression and protein abundance—can be incorporated as intermediate traits that directly reflect immune activity (99). This approach enhances the identification of functional variants regulating antiviral responses. In parallel, selection signature analyses suggest that immune-related loci have been shaped by long-term evolutionary pressures, although their specific roles in ALV immunity remain to be fully elucidated (100–102). However, linking these signals to ALV-specific immune responses remains challenging. Together, these findings underscore the importance of integrating genomic variation with functional immune readouts to better define the genetic basis of host immunity.

### 4.2 Transcriptomics reveals dynamic immune response during ALV infection

Transcriptomic analyses capture how host immune responses are dynamically activated and regulated during ALV infection (**Figure 4B**). By profiling genome-wide expression changes, transcriptomics provides a systems-level view of host–virus interactions. ALV infection induces robust activation of innate immune pathways, particularly those mediated by PRRs and interferon (IFN) signaling (103–105). A large number of interferon-stimulated genes (ISGs) are upregulated and contribute to antiviral defense, while non-coding RNAs further modulate immune signaling and viral replication (106, 107). In addition, single-cell transcriptomic studies reveal pronounced immune heterogeneity, characterized by the coexistence of antiviral effector cells and immunosuppressive cell populations (108–110). Beyond gene expression changes, ALV infection also alters RNA processing, including alternative splicing, which can reshape immune signaling pathways and facilitate viral immune evasion (111, 112). This dynamic balance between immune activation and suppression represents a hallmark of ALV infection and contributes to viral persistence and incomplete clearance.

Overall, transcriptomics demonstrates that ALV infection triggers multilayered immune response, linking transcriptional regulation to antiviral outcomes and providing important molecular phenotype of host immunity.

### 4.3 Proteomics provides insights into host–virus interactions during ALV infection

While transcriptomics reveals regulatory changes at the RNA level, proteomics provides direct insights into the functional molecules that execute host responses and regulate viral replication. As the ultimate effectors of cellular processes, proteins play central roles in both antiviral defense and viral propagation(**Figure 4C**).

Compared with genomic and transcriptomic studies, proteomic investigations of ALV remain relatively limited. Nevertheless, available evidence suggests that host proteins participate in multiple stages of the viral life cycle. Proteomic analyses have demonstrated that host proteins can be incorporated into ALV particles during virion assembly and budding, indicating potential roles in viral maturation, infectivity, and virus–host interactions (113). Although direct evidence in ALV remains limited, studies of moloney murine leukemia virus (MMLV), another retrovirus, have shown that extracellular vesicles carry host proteins capable of modulating intercellular communication and the immune microenvironment during infection (114). Insights from other viral systems further highlight the value of proteomics in dissecting host–virus interactions. In many RNA viruses, including influenza virus, hepatitis C virus, and coronaviruses, proteomic approaches have revealed extensive interactions between viral components and host proteins involved in translation, intracellular trafficking, metabolism, and signal transduction (115, 116). These host factors are frequently co-opted by viruses to facilitate replication, assembly, and dissemination. Moreover, post-translational modifications (PTMs), such as phosphorylation, ubiquitination, and acetylation, constitute an additional regulatory layer through which viruses remodel host signaling pathways, modulate antiviral responses, and establish favorable conditions for infection (96, 117–119).

Despite these advances, the global landscape of protein–protein interactions between ALV and host factors remains incompletely characterized, highlighting the need for further integrative analyses to define functional interaction networks underlying viral infection.

### 4.4 Epigenomics and epitranscriptomics: regulatory layers of immune modulation and viral persistence

Epigenomic and epitranscriptomic provide a key insights into the molecular basis of host and virus interaction by reshaping chromatin states and RNA modifications which modulate antiviral responses, viral persistence, and immune evasion (120–122). These processes regulate gene expression without altering DNA sequence, thereby linking genetic variation to functional immune outcomes (**Figure 4D**).

ALV infection can alter DNA methylation and histone modification patterns, thereby modulating key immune signaling pathways such as the MDA5–TBK1 axis and interferon responses (123–125). These changes contribute to immune suppression and facilitate viral persistence. As an integrating retrovirus, ALV directly reshapes the host epigenetic landscape through insertion into the genome, which can disrupt gene regulation and promote oncogenic transformation (126–129). This highlights the dual role of epigenetic mechanisms in both antiviral defense and viral pathogenesis.

At the RNA level, epitranscriptomic modifications, particularly N6-methyladenosine (m6A), represent a dynamic and reversible layer of RNA regulation that influences RNA stability, localization, transport, and translation (130, 131). In ALV-J-induced chicken liver tumors, approximately 83% of differentially expressed transcripts exhibit one to two m6A peaks within coding regions, and these modifications may influence the expression of lncRNAs and miRNAs, thereby indirectly regulating viral replication and tumorigenesis (132, 133). Together, epigenomic and epitranscriptomic mechanisms form a dynamic regulatory interface through which ALV modulates host immunity and maintains persistent infection.

### 4.5 CRISPR-based screening bridges multi-omics discovery and functional immunology

While multi-omics approaches generate extensive lists of candidate genes, functional validation remains a major challenge. CRISPR-based screening provides an efficient strategy for systematically interrogating gene function by enabling large-scale perturbation of host genes (134). While its application in ALV research is still limited, studies in related avian viruses, such as avian influenza virus and Newcastle disease virus, have demonstrated its feasibility in identifying critical immune regulators (135, 136). The non-cytolytic and persistent nature of ALV infection presents additional challenges for screening design, but emerging strategies with recombinant fluorescent virus are beginning to overcome these limitations (75). Beyond knockout approaches, CRISPR interference and CRISPR activation systems further enable the dissection of gene regulatory networks and dosage-dependent effects (137–139).

Importantly, CRISPR-based approaches serve as a bridge between multi-omics discovery and mechanistic understanding, allowing the identification of causal regulators within complex host–virus interaction networks (**Figure 4E**). This integrative framework is essential for translating omics-derived signatures into functional insights and advancing the study of viral immunology.

## 5 Challenges and future perspectives

### 5.1 Vaccine development

Compared with many acute viral infections, progress in vaccine development against ALV remains limited, and no broadly effective commercial vaccines are currently available. This is largely due to the high genetic variability of ALV and its replication strategy as an integrating retrovirus (140).

Substantial antigenic diversity, particularly within the env gene encoding gp85, results in pronounced differences among viral subgroups and poor cross-protective immunity (141). In addition, ALV infection often induces immune tolerance or immunosuppression, especially following embryonic or early-life exposure, thereby impairing the establishment of effective antiviral immune responses. The predominance of vertical transmission further complicates control, as conventional vaccination strategies are insufficient to block transmission through eggs.

Current vaccine strategies, including subunit and recombinant gp85-based vaccines, can elicit neutralizing antibodies but fail to provide consistent protection. DNA vaccines show limited expression efficiency in avian systems, while mRNA vaccines remain constrained by delivery challenges and cost (142). Collectively, these limitations highlight that effective control of ALV cannot rely solely on pathogen-targeted strategies, and underscore the need to better understand host immune regulation during infection (143, 144).

### 5.2 Rethinking antiviral strategies: from resistance to immune-mediated tolerance

For persistent viruses such as ALV, complete resistance is difficult to achieve and may impose strong selective pressure on viral evolution. Increasing attention has therefore shifted toward disease tolerance, defined as the ability of the host to maintain physiological function despite infection (145–147). In the context of ALV, tolerance is closely linked to immune regulation, including the ability to control viral replication while avoiding excessive immune activation and immunopathology. This balance is shaped by complex interactions among innate immune sensing pathways, cytokine signaling, and cellular processes such as apoptosis and metabolism. From an immunological perspective, this shift emphasizes not only antiviral defense but also immune homeostasis, suggesting that host–virus interactions should be understood as a dynamic equilibrium rather than a unidirectional antiviral response.

### 5.3 Phenotypic bottlenecks and the need for immune-relevant molecular readouts

A major obstacle in dissecting host–ALV interactions lies in the lack of precise and dynamic phenotypic evaluation systems. Traditional indicators, such as pathological lesions or viremia levels, are often retrospective, variable, and insufficient to capture the early and subclinical stages of infection (148–150). Although molecular diagnostic tools, including ELISA and qRT-PCR, are widely used, they primarily reflect viral presence rather than the underlying immune state. Given that ALV infection is characterized by persistent and immunosuppressive features, understanding host responses requires more sensitive and mechanistically informative indicators (151). Molecular phenotypes, such as gene expression profiles, protein abundance, and epigenetic modifications, provide a direct and quantitative readout of immune activation states and regulatory dynamics (150). These features capture the dynamic responses of immune pathways, including interferon signaling, PRR activation, and immune cell differentiation. In addition, recent studies in poultry have demonstrated the utility of integrating multi-omics data to identify molecular signatures associated with economically important traits, such as growth performance, survival, and resistance to bacterial infections (152–154). However, their application in ALV research remains limited, particularly in terms of standardized datasets and multi-tissue, time-resolved analyses. Establishing comprehensive molecular phenotype frameworks will therefore be critical for linking immune responses to genetic variation and for improving the resolution of host–virus interaction studies (100).

### 5.4 Toward integrative multi-omics dissection of host–virus interaction networks

Given the multi-layered nature of host immune responses to ALV infection, future studies should move beyond isolated analyses and adopt integrative frameworks that reconstruct host–virus interaction networks across molecular layers. By combining genomics, transcriptomics, proteomics, and epigenomics, it becomes possible to map how genetic variation, transcriptional regulation, protein function, and chromatin dynamics collectively shape antiviral immunity and viral persistence. Within this framework, molecular phenotypes serve as critical intermediates that link genetic variation to immune function, enabling the identification of regulatory nodes controlling interferon responses, immune suppression, and viral replication (155, 156). However, association alone is insufficient to establish causality. CRISPR-based perturbation systems provide a complementary approach by enabling systematic functional interrogation of candidate genes and pathways. Through targeted gene disruption or activation, these systems allow direct testing of how specific host factors regulate immune signaling and viral life cycles. Together, the integration of multi-omics profiling with functional perturbation establishes a systems immunology framework for dissecting ALV–host interactions. Such an approach not only refines our understanding of the regulatory logic underlying antiviral immunity but also provides a conceptual basis for translating mechanistic insights into disease control strategies (155).

### 5.5 Limitations of this review

Several limitations of this review should be acknowledged. First, the discussion is primarily focused on ALV infection in chickens, as most available studies have been conducted in commercial and indigenous chicken populations. Consequently, host–virus interactions in other avian species remain insufficiently explored (157). Second, although this review emphasizes the potential of integrative multi-omics approaches, most currently available studies rely on single-omics datasets. Therefore, the proposed multi-omics framework is largely based on the integration of findings from independent studies rather than fully integrated datasets generated within a unified experimental design. Despite these limitations, we believe that synthesizing current evidence across multiple omics provides a useful perspective for understanding ALV–host interactions and highlights important directions for future research.

## 6 Conclusion

In summary, ALV infection represents a complex immunological process shaped by the dynamic interplay between viral persistence and host immune regulation. Although substantial progress has been made in identifying individual host factors and signaling pathways, current knowledge remains fragmented and insufficient to capture the coordinated, multi-layered nature of antiviral responses. Increasing evidence indicates that ALV pathogenesis is governed not by isolated genes or pathways, but by interconnected regulatory networks spanning genomic variation, transcriptional reprogramming, protein interactions, and epigenetic modifications. These layers collectively determine the balance between viral replication, immune activation, and immune evasion, ultimately shaping infection outcomes. Against this backdrop, multi-omics approaches provide a powerful framework to reconstruct the dynamic landscape of host–virus interactions, enabling the identification of key regulatory nodes that cannot be resolved through single-layer analyses. The incorporation of molecular phenotypes further bridges genetic variation and functional immune states, offering a more refined perspective on host responses to infection. However, translating these associations into mechanistic insight requires systematic functional interrogation. In this regard, CRISPR-based perturbation strategies offer an effective means to define causal relationships within host–virus interaction networks and to validate key regulatory factors. Collectively, the integration of multi-omics profiling with functional genomics establishes a systems-level framework for dissecting ALV-host interactions. This shift from descriptive to mechanism-driven research not only advances our understanding of viral immunology in poultry, but also provides a conceptual basis for exploring host-directed strategies to regulate immune responses and control persistent viral infections. Ultimately, such integrative approaches may offer broader insights into the fundamental principles governing host-pathogen interactions across species.
